# Novel Insights Into the Bioactive Profile and Therapeutic Potentials of Indonesian *Annona muricata* Leaves

**DOI:** 10.1002/cbdv.202501606

**Published:** 2025-08-19

**Authors:** Sara Oufquir, Fatimazahra Agouram, Hamid Kabdy, Mehdi Ait Laaradia, Khadija Oubella, Baslam Abdelmoniim, Aboufatima Rachida, Stefania Garzoli, Abderrahman Chait

**Affiliations:** ^1^ Department of Biology, Laboratory of Pharmacology, Neurobiology, Anthropobiology and Environment, Faculty of Sciences Semlalia Cadi Ayyad University Marrakech Morocco; ^2^ Laboratory of Génie Biologique, Faculty of Sciences and Technics Sultan Moulay Slimane University Béni Mellal Morocco; ^3^ Department of Chemistry and Technologies of Drug Sapienza University Rome Italy

**Keywords:** *Annona muricata*, anti‐inflammatory, antinociceptive, antioxidant activity, aqueous extract, graviola

## Abstract

This study investigates, for the first time, the comprehensive pharmacological profile of the aqueous extract of *Annona muricata* L. (graviola) leaves from Indonesia, focusing on its antioxidant, antinociceptive, and anti‐inflammatory properties, alongside its acute toxicity and phytochemical composition. The extract, obtained through cold maceration, was rich in phenolic compounds, including gallic acid, catechin, quercetin, and rutin, as revealed by phytochemical analysis. It exhibited strong antioxidant capacity in both 2,2‐diphenyl‐1‐picrylhydrazyl (DPPH) and ferric‐reducing antioxidant power (FRAP) assays. Acute toxicity testing indicated no adverse effects at doses up to 5000 mg/kg, confirming its safety. Notably, the extract produced significant antinociceptive effects in hot plate, acetic acid–induced writhing, and formalin tests and exerted marked anti‐inflammatory activity in both xylene‐induced ear edema and carrageenan‐induced paw edema models, comparable to the standard drug indomethacin. These findings not only support the traditional use of *A. muricata* in pain and inflammation relief but also provide novel evidence of its therapeutic potential as a safe, natural source of bioactive compounds with multiple pharmacological effects.

## Graphical abstract

Pharmacological investigation of the aqueous extract of Annona muricata L. leaves from Indonesia.

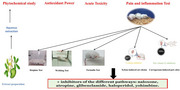

## 1 Introduction

The global burden of chronic inflammatory, cardiovascular, and pain‐related conditions remains a major concern due to their high prevalence and long‐term impact on health systems [1, 2]. These conditions are often driven by prolonged oxidative stress and inflammation, which contribute to progressive tissue damage and functional decline [3]. Although conventional therapies have improved patient outcomes, especially in early disease stages, their long‐term use is frequently associated with adverse effects, limited efficacy in advanced stages, resistance to current treatments, and high treatment costs [4, 5]. As a result, managing advanced conditions remains challenging and has led to growing interest in the search for safer, more affordable, and multifunctional therapeutic alternatives.

Medicinal plants have played a central role in healthcare for millennia and continue to be a cornerstone of primary health systems worldwide. According to the World Health Organization (WHO), around 80% of the global population still relies on plant‐based remedies for their primary healthcare needs [6]. These plants, rich in structurally diverse bioactive compounds, particularly phenolics, flavonoids, and tannins, have demonstrated broad‐spectrum therapeutic potential. They are especially valued for their antioxidant, anti‐inflammatory, and analgesic properties, which are crucial in mitigating oxidative stress and inflammation, key mechanisms underlying the development and progression of many chronic diseases [7].

Among these medicinal plants, *Annona muricata* L. (commonly known as graviola or soursop), a tropical species from the Annonaceae family native to South America and the Caribbean [8], is traditionally used to treat a wide range of ailments. These include gastrointestinal disorders, parasitic infections, joint pain, inflammatory diseases [9], respiratory conditions [10], and fever [9]. Ethnobotanical data also support its use as a sedative [11], insecticide [12], antiparasitic [13], and in the treatment of malaria [14], as well as liver and kidney conditions [15].

Pharmacologically, *A. muricata* has garnered increasing interest due to its unique profile of bioactive compounds, particularly acetogenins. These compounds have shown hypoglycemic [16], hypotensive [17], antinociceptive, and anticancer activities [18]. Furthermore, the plant exhibits notable antioxidant activity, making it effective in neutralizing free radicals and protecting against oxidative damage associated with cardiovascular and neurodegenerative diseases [18]. Its ability to alleviate pain and reduce both acute and chronic inflammation further supports its potential in managing various chronic conditions [10, 19].

Despite its widespread traditional use and the growing number of pharmacological studies, scientific evaluation of *A. muricata* remains incomplete. Most research has focused on isolated compounds or ethanolic extracts, whereas aqueous extracts, the form most commonly used in traditional medicine, have received comparatively less attention. Additionally, although some studies have suggested low toxicity, there remains a need for comprehensive investigations into its acute toxicity to ensure safety, particularly in the context of prolonged use or higher dosages [13, 14, 20, 21]. A thorough chemical analysis, coupled with toxicological and pharmacological studies using standardized experimental models, is essential for validating its traditional applications and elucidating its full therapeutic potential.

Therefore, this study aims to address these gaps by evaluating the antioxidant, antinociceptive, and anti‐inflammatory activities of the aqueous extract of *A. muricata* (AEAM) leaves, alongside its acute toxicity profile and phytochemical composition. By integrating traditional knowledge with scientific evidence, we seek to highlight *A. muricata* as a promising natural source of therapeutic compounds for the prevention and treatment of chronic inflammatory and pain‐related disorders.

## 2 Results and Discussion

### 2.1 Phytochemical Study

The preliminary phytochemical screening of AEAM revealed the presence of flavonoids, tannins, leucoanthocyanins, terpenes, and steroids and the absence of saponins, anthocyanins, and quinones. According to our quantitative colorimetric analysis results, we have distinguished that the *A. muricata* extract has a large amount of polyphenols (16.8 ± 0.54 mg GAE/g extract), flavonoids (6.2 ± 0.27 mg QE/g extract), and condensed tannins (2.8 ± 0.12 mg GAE/g extract) (Table 1).

**TABLE 1:** Content of total polyphenols, flavonoids, and condensed tannins.

	*Annona muricata* leaves
Total polyphenols (mg GAEeq/g MS)	16.8 ± 0.54
Flavonoids (mg QEeq/g MS)	6.2 ± 0.27
Condensed tannins (mg CAEeq/g MS)	2.8 ± 0.12

*Note*: The results are expressed in mg/mL and presented as the mean ± SEM.

### 2.2 Phytochemical Characterization by High‐Performance Liquid Chromatography (HPLC)

**FIGURE 1:**
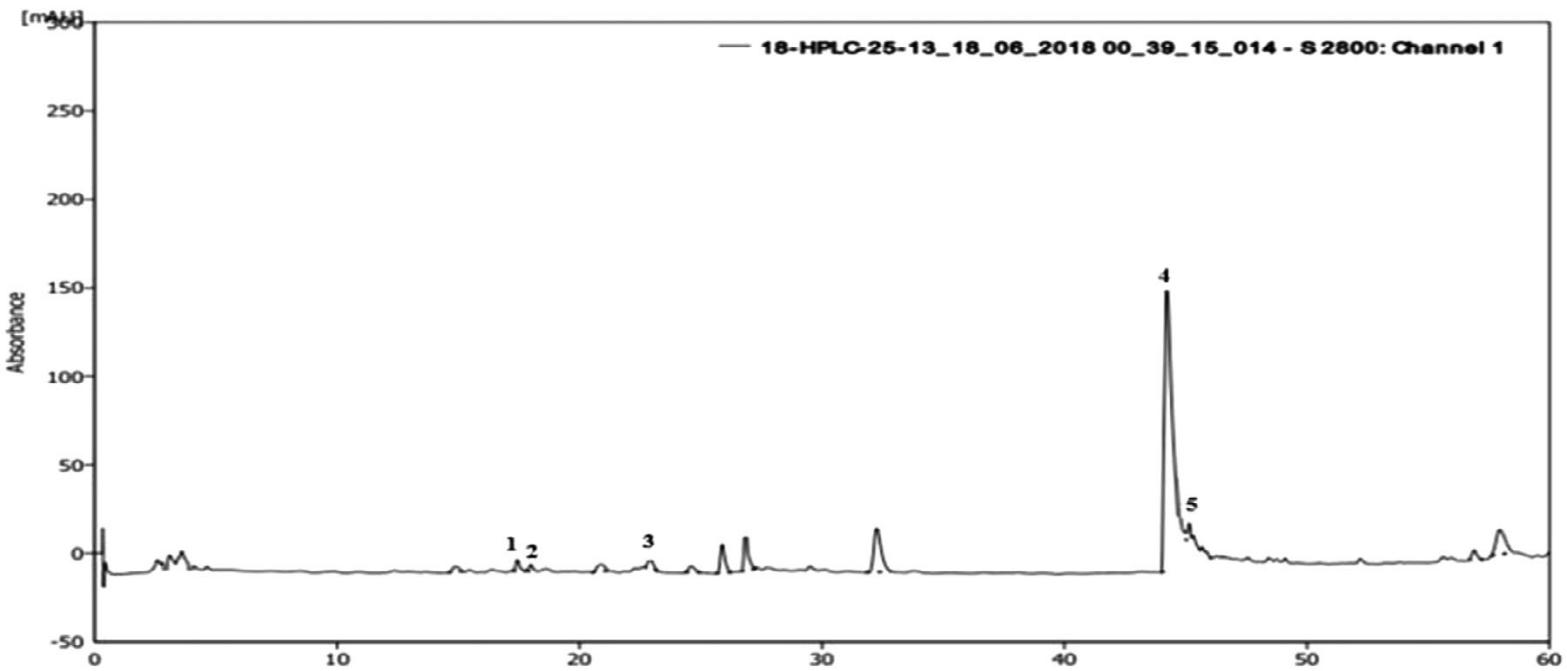
HPLC chromatogram of the main phenolic compounds identified in the aqueous extract of *Annona muricata* leaves, 1: syringic acid; 2: tyrosol; 3: rutin; 4: catechin; 5: quercetin. HPLC, high‐performance liquid chromatography.

An HPLC analysis of *A. muricata* extract revealed a chromatogram with four main components, including flavonoids and phenolic compounds. On the basis of the retention times (min) of standard compounds, the identified polyphenols were syringic acid, tyrosol, rutin, and quercetin (Figure 1). Catechin was present in the highest concentration (28.32 mg GAE/100 g DM), followed by quercetin (14.48 mg GAE/100 g DM), whereas rutin, syringic acid, and tyrosol were found at concentrations of 12.39 mg GAE/100 g DM, 12.31 mg GAE/100 g DM, and 12.18 mg GAE/100 g DM, respectively.

### 2.3 Antioxidant Activity

#### 2.3.1 Trapping of the 2,2‐Diphenyl‐1‐Picrylhydrazyl (DPPH) Radical

*A. muricata* extract aqueous showed a significant power with an intermediate value between butylated hydroxytoluene (BHT) and vitamin C because it is of the order of 0.210 ± 0.030 mg/mL (Table 2).

**TABLE 2:** Anti‐radical activity of 2,2‐diphenyl‐1‐picrylhydrazyl (DPPH) from the aqueous extract of *Annona muricata*.

	DPPH (IC_50_ mg/mL)
*Annona muricata* leaves	0.210 ± 0.030
BHT	0.350 ± 0.002
Vitamin C	0.140 ± 0.003

*Note*: The results are expressed in mg/mL and presented as mean ± SEM.

Abbreviation: BHT, butyl hydroxytoluene.

#### 2.3.2 FRAP (Ferric‐Reducing Antioxidant Power) Iron Reduction Test and β‐Carotene Bleaching Test

The reducing activity results clearly show that the AEAM has the reducing power of ferric ions (Table 3).

**TABLE 3:** Reducing power and β‐carotene bleaching of aqueous extract of *Annona muricata*.

	Reducing power (IC_50_ mg/mL)	β‐Carotene bleaching (IC_50_ mg/mL)
*Annona muricata* leaves	0.81 ± 0.012	0.122 ± 0.410
Quercetin	0.07 ± 0.001	2.621 ± 0.020
BHT	0.12 ± 0.008	0.070 ± 0.001

*Note*: The results are expressed in mg/mL and presented as mean ± SEM.

Abbreviation: BHT, butyl hydroxytoluene.

The examination of the results of inhibition of β‐carotene oxidation shows that the extract has an antioxidant power that largely exceeds those of the reference substances. In fact, the extract has a high IC_50_ value of 0.122 ± 0.410 mg/mL, but it remains largely lower than that of quercetin, which has an IC_50_ value of around 2.621 ± 0.020 mg/mL (Table 3).

### 2.4 Acute Toxicity

#### 2.4.1 Estimation of LD_50_

**FIGURE 2:**
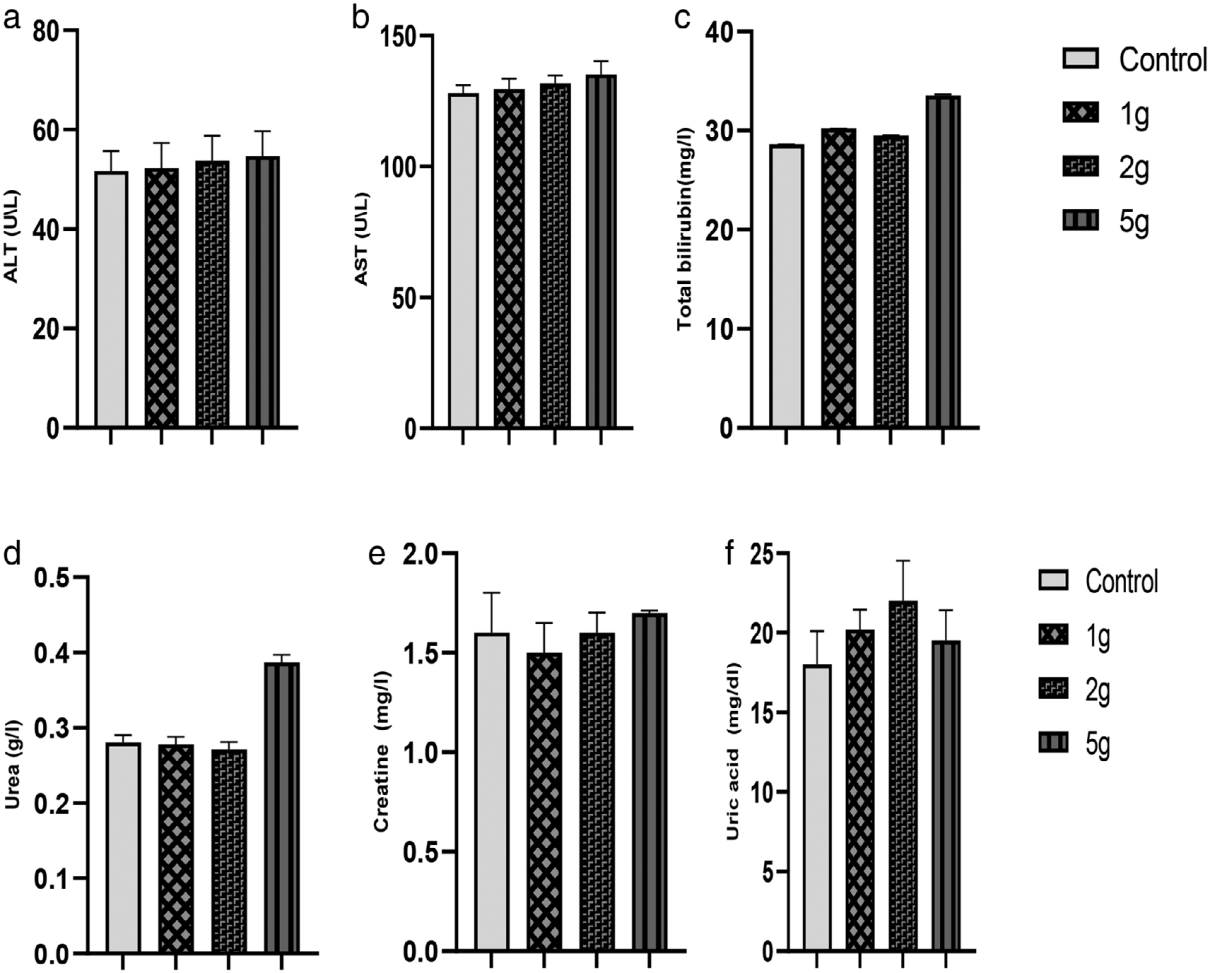
Effect of *Annona muricata* aqueous extract on liver and renal functions: ALAT (alanine aminotransferase) (a), ASAT (aspartate aminotransferase) (b), total bilirubin (c), urea (d), creatinine (e), and uric acid (f). Results are expressed as mean ± SEM (*n* = 5 per group). Data were analyzed using one‐way ANOVA followed by Tukey's post hoc test. No statistically significant differences were observed among the groups (*p* > 0.05).

In this study, oral administration of *A. muricata* extract at doses ranging from 1 to 5 g/kg had no observable effect on the behavioral responses of the animals (Figure 2). Furthermore, no allergic reactions and no mortality were observed during the 48‐h period following administration, nor during the subsequent 7‐day observation period. These results suggest that the extract has a low toxicity profile, with an LD_50_ greater than 5 g/kg b.w.

### 2.5 Hematological, Biochemical, and Histopathological Analysis

**FIGURE 3:**
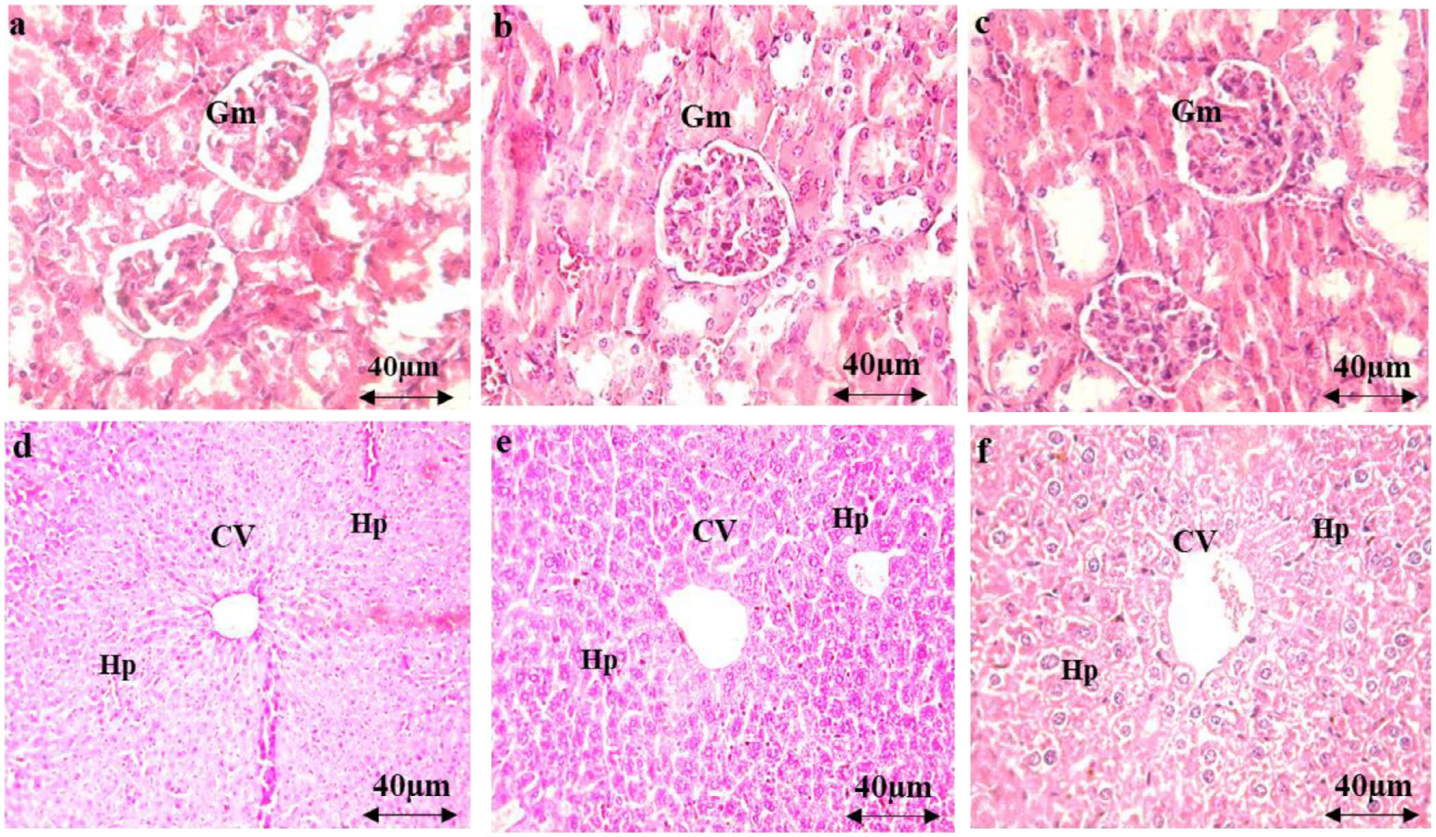
Histopathological examinations of organs (liver and kidneys) in acute toxicity: kidney tissue—control (a), AEAM 250 mg/kg (b), and AEAM 500 mg/kg (c); liver tissue—control (d), AEAM 250 mg/kg (e), and AEAM 500 mg/kg (f). Sections were stained and colored with hematoxylin and eosin (×200). CV: central vein; Gm: glomerulus; hp: hepatocyte.

The administration of AEAM did not affect biochemical parameters (urea, uric acid, creatinine, aspartate aminotransferase [ASAT], alanine aminotransferase [ALAT], and total bilirubin) at any of the tested doses (Figure 3), with only mild toxicity observed at the 5 g/kg dose. Microscopic examination of renal and hepatic tissues revealed normal histological features, comparable to those of the control group, indicating that oral administration of the AEAM did not cause any histopathological alterations.

#### 2.5.1 Hotplate Test

**FIGURE 4:**
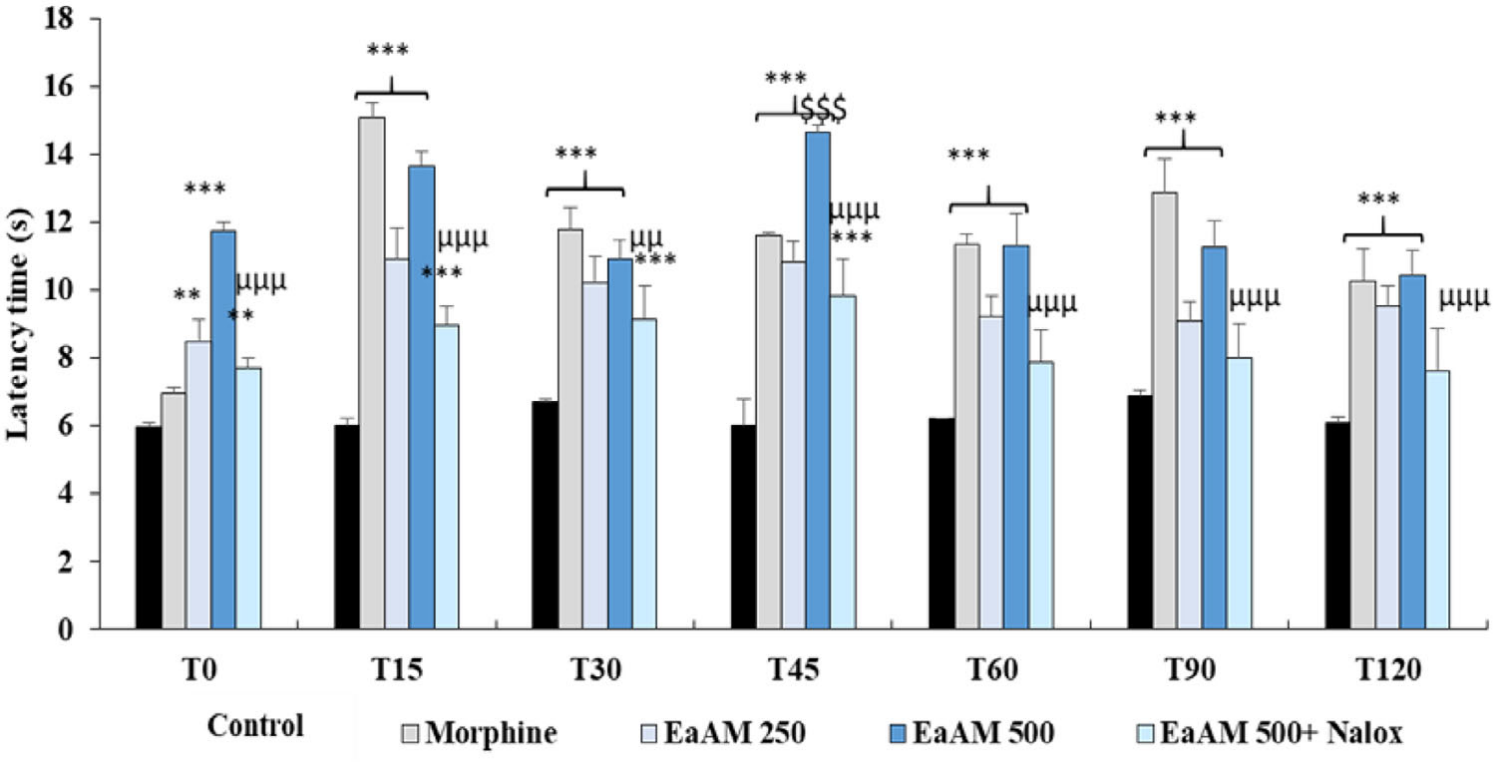
Antinociceptive effect of aqueous extract of *Annona muricata* (AEAM) leaves with Naloxone pretreatment on latency time in the hot plate test (*n* = 5). Nalox (naloxone; 2 mg/kg). Results are presented as mean ± SEM. ***p < 0.001 versus control, ^μμμ^ *p* < 0.001 versus AEAM 500; ^$$$^ *p* < 0.001 versus morphine (ANOVA followed by a post hoc test: Tukey).

The results presented demonstrate a significant antinociceptive effect of the extract, which becomes apparent within the first few minutes after administration. The AEAM exhibited a relatively rapid and moderate antinociceptive effect, reaching its peak at 45 min and remaining detectable, though gradually declining, at both tested doses (250 and 500 mg/kg) (Figure 4).

In comparison with morphine, *A. muricata* exhibited a comparable, and even superior, antinociceptive effect (*p* < 0.001). Furthermore, co‐administration of naloxone with the highest tested dose of the aqueous extract significantly inhibited its antinociceptive activity (500 mg/kg; *p* < 0.001) (Figure 4).

#### 2.5.2 Writhing Test

**FIGURE 5:**
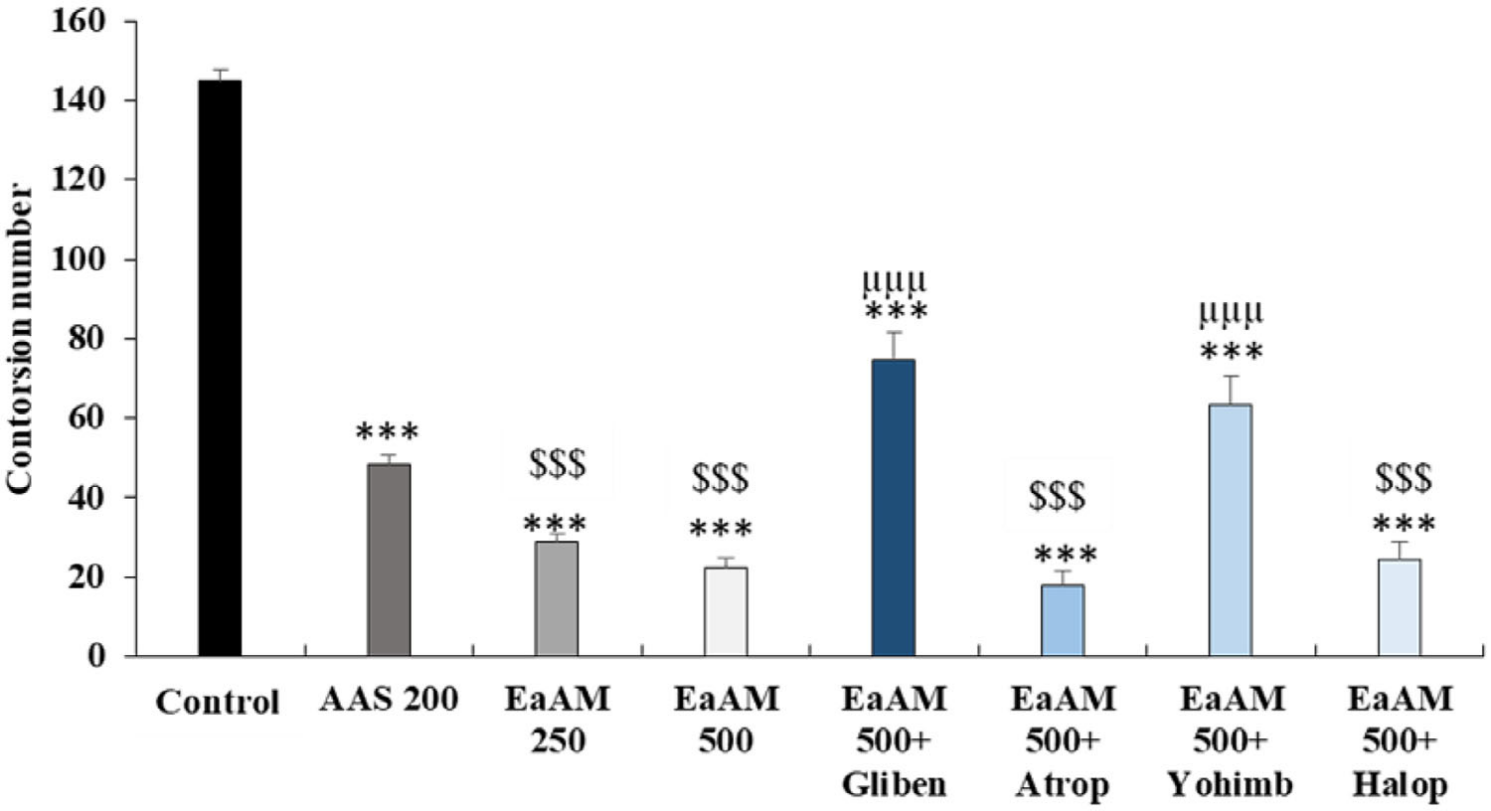
Antinociceptive effect of the aqueous extract of *Annona muricata* leaves (AEAM) with pretreatment with glibenclamide, atropine, yohimbine, and haloperidol on the number of contortions (*n* = 5); Gliben (glibenclamide: 10 mg/kg); Atrop (atropine: 5 mg/kg); Yohimb (yohimbine: 3 mg/kg); Halop (haloperidol: 5 mg/kg). Results are presented as mean ± SEM. ***p < 0.001 versus control, ^μμμ^ *p* < 0.001 versus AEAM 500; ^$$$^ *p* < 0.001 versus ASA (acetylsalicylic acid; 200 mg/kg) (ANOVA followed by a post hoc test: Tukey).

The results presented in Figure 5 demonstrate that AEAM extract significantly inhibits the number of contortions and the pain caused by the injection of acetic acid (250 and 500 mg/kg; *p* < 0.001). Furthermore, the injection of glibenclamide significantly antagonized the effect of *A. muricata* (500 mg/kg; *p* < 0.001). Regarding atropine, yohimbine significantly inhibited the effect of AEAM (500 mg/kg; *p* < 0.001). In contrast, haloperidol had no significant effect on the activity of the extract (Figure 5).

#### 2.5.3 Formalin Test

**FIGURE 6:**
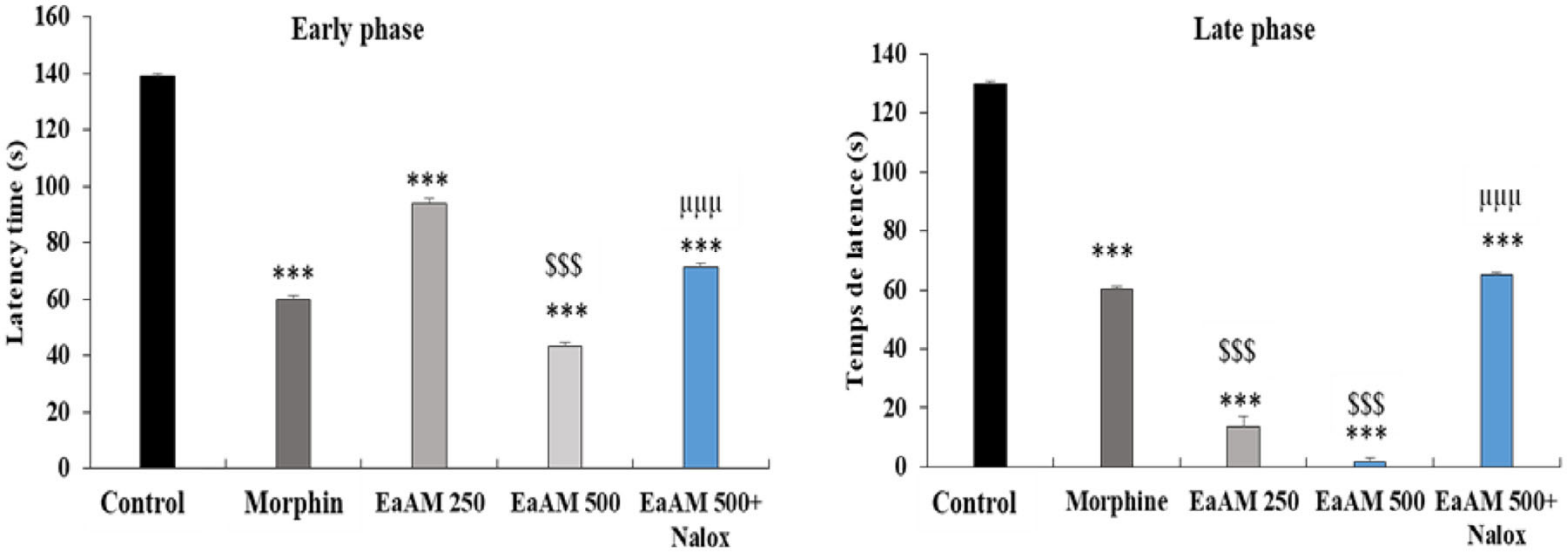
The antinociceptive effect of the aqueous extract of *Annona muricata* leaves (AEAM) was evaluated in the formalin test in the presence of the opioid antagonist naloxone (Nalox; 2 mg/kg). The antagonist naloxone (2 mg/kg) was administered. The results are presented as mean ± SEM. Statistical significance was determined by one‐way ANOVA with Tukey's post hoc test. ***p < 0.001 versus control, ^μμμ^p < 0.001 versus AEAM 500; ^$$$^ *p* < 0.001 versus morphine. The data were analyzed using a one‐way analysis of variance (ANOVA) followed by a post hoc test (Tukey).

In the initial stages of the study, it was observed that AEAM exhibited a significant, dose‐dependent antinociceptive effect (*p* < 0.001). This effect was found to be comparable to that of morphine in some cases. Notably, the extract demonstrated a level of activity that significantly exceeded that of morphine at a dose of 500 mg/kg (*p* < 0.001) (Figure 6).

During the late phase, a highly significant effect was observed for the aqueous extract, which significantly exceeded that of the reference molecule, morphine (*p* < 0.001) (Figure 6). Furthermore, the addition of naloxone significantly antagonized the antinociceptive effect of the tested extract, both during the early phase and during the late phase.

#### 2.5.4 Xylene‐Induced Ear Edema

The results of the inflammatory response following xylene application to the ear are presented in Table 4. A significantly greater increase in ear weight was observed in the control group compared to the treatment groups. Specifically, AEAM at doses of 250 mg/kg (37.29%) and 500 mg/kg (59.48%) showed significant inhibition of xylene‐induced ear edema in mice compared to the control group (*p* < 0.05). Notably, indomethacin (10 mg/kg) exhibited a pronounced anti‐inflammatory effect, reducing ear edema by 52.95% (*p* < 0.001). These findings highlight the effectiveness of AEAM in significantly reducing xylene‐induced inflammation.

**TABLE 4:** The effect of aqueous extract of *Annona muricata* (AEAM) (250 and 500 mg/kg) and indomethacin in the xylene‐induced ear edema test in mice.

Treatment	Ear edema (mg)	Inhibition (%)
Control	30.46 ± 10.22	—
Indomethacin 10 mg/kg	14.33 ± 1.11^c^	52.95
AEAM 250 mg/kg	19.1 ± 1.27^a^	37.29
AEAM 500 mg/kg	12.34 ± 1.01^b^	59.48

*Note*: The data are expressed as mean ± SEM; *n* = 6; the superscript “a” indicates a *p* value <0.05; “b” indicates a *p* value <0.01; “c” indicates a *p* value <0.001.

#### 2.5.5 Histological Examination

**FIGURE 7:**
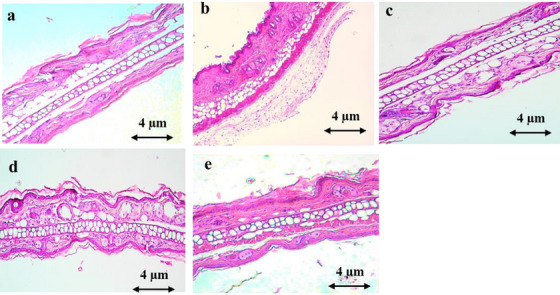
Tissue sections indicating the swelling of the ear caused by xylene treatment: (a) the normal ear; (b) model control group induced by xylene; (c) the treatment with diclofenac (10 mg/kg); (d) the treatment with AEAM (250 mg/kg); (e) the treatment with AEAM (500 mg/kg); (HE ×100). Scale bar = 4 µm.

Figure 7 demonstrates that xylene application resulted in ear lesions, characterized by a notable increase in epidermal thickness, edema, and infiltration of polymorphonuclear leukocytes. However, oral treatment with AEAM extract (250 and 500 mg/kg) and diclofenac (10 mg/kg) significantly mitigated these effects.

#### 2.5.6 Carrageenan‐Induced Edema Test

Paw edema induced by carrageenan injection was significantly reduced by AEAM at all tested doses (*p* < 0.001) (Table 5). AEAM exhibited a rapid onset of anti‐inflammatory action, showing an effect comparable to that of ASA from the first hour (*p* < 0.001) at a dose of 500 mg/kg. Furthermore, at the fifth hour, AEAM demonstrated a highly significant anti‐inflammatory effect, comparable in magnitude to ASA. At the highest dose, inhibition of paw edema reached approximately 60% (*p* < 0.001) (Table 5).

**TABLE 5:** The effect of an aqueous extract of *Annona muricata* (AEAM) on edema induced in mice by carrageenan injection was investigated.

	Volume of edema			Inhibition (%)		
	1 h	3 h	5 h	1 h	3 h	5 h
Negative control	0.19 ± 0.03	0.30 ± 0.03	0.32 ± 0.01	—	—	—
Positive control (ASA)	0.10 ± 0.01^***^	0.12 ± 0.02^***^	0.10 ± 0.03^***^	47.00	60.00	68.75
AEAM 250 mg/kg	0.13 ± 0.02^***^	0.15 ± 0.03^***^	0.14 ± 0.02^***^	31.57	50.00	56.25
AEAM 500 mg/kg	0.11 ± 0.01^***^	0.14 ± 0.02^***^	0.12 ± 0.01^***^	42.10	53.33	62.50

*Note*: Results are presented as mean ± SEM.

Abbreviation: ASA, acetylsalicylic acid.

*** *p* < 0.001 versus negative control.

*A. muricata*, commonly known as graviola, is garnering increasing interest due to its multiple therapeutic properties, which are attributed to its richness in secondary metabolites and its low toxicity. This tropical plant has traditionally been used to treat various ailments, including inflammatory and painful conditions [14, 22].

Pain and inflammation, two processes often interconnected, highlight its antioxidant potential, a key factor in the prevention of these conditions. The interest in this plant also lies in its low level of toxicity, ensuring safe use at appropriate doses.

Phytochemical investigation of the extract of *A. muricata* revealed a richness in various bioactive compounds. Among these, flavonoids, tannins, and leucoanthocyanins are present, whereas anthocyanins and saponins were not detected. These results confirm observations from numerous studies that have highlighted the presence of chemical classes, such as alkaloids, terpenoids, flavonoids, and tannins in leaf extracts, whether in aqueous or ethanolic solution [23, 24]. This complex chemical composition endows *A. muricata* with its antioxidant, analgesic, and anti‐inflammatory properties.

Moreover, acetogenins, another major group of bioactive compounds, were successfully identified in the leaves of *A. muricata*. Zubaidi et al. [10] employed HPLC, nuclear magnetic resonance (NMR), and Fourier‐transform infrared (FT‐IR) spectroscopy to characterize these compounds. These acetogenins, recognized for their anticancer properties, play an important role in the therapeutic effects of the plant.

However, the absence of quinones in the studied extract is notable. These compounds, although frequently present in fruits and vegetables, play a crucial role in electron transport and combating oxidative stress [25]. They are also responsible for the browning reaction of cut or damaged fruits and vegetables [26]. Contrary to our results, Naik and Sellappan [27, 29] reported the presence of quinones in *A. muricata*. These discrepancies may be related to differences in extraction methods or the environmental conditions of the studied plants.

From a quantitative perspective, the leaves of *A. muricata* exhibit a high content of polyphenols, flavonoids, and condensed tannins. According to Nguyen et al. [28, 29], the total phenolic content (TPC) and total flavonoid content (TFC) in the ethanolic extract of the leaves reaches 609.08 ± 5.82 mg GAE/g and 209.52 ± 1.88 mg QE/g, respectively. Additionally, HPLC analysis, a more sophisticated method, revealed the presence of syringic acid, tyrosol, and rutin in *A. muricata*. Furthermore, graviola is distinguished by a high level of catechin and a notable amount of quercetin. Thirty‐seven phenolic compounds have been identified in *A. muricata* [30], including quercetin [29] and gallic acid [31].

It was found that the ethanolic extract of the stem bark of *A. muricata* contained alkaloids, saponins, tannins, flavonoids, phenolic compounds, and glucosides. Treatment with this extract (200 mg/kg b.w.) significantly altered serum enzyme levels and oxidants, bringing them close to normal in CCl_4_‐treated rats. In vivo and in vitro studies on the ability of this bark extract to rapidly scavenge free radicals yielded positive results, suggesting that the possible mechanism of the biological activities of the extract could be related to free radical scavenging due to the presence of polyphenols. The plant extract possesses antioxidant and anti‐lipid peroxidation effects [32].

The antioxidant activity of *A. muricata* is closely linked to its chemical composition, particularly the presence of polyphenols, flavonoids, and condensed tannins. These compounds primarily act by scavenging free radicals, a key mechanism to counter oxidative stress, which is the origin of many inflammatory and degenerative pathologies [33].

The tests conducted revealed a remarkable antioxidant effect of graviola, sometimes exceeding that of reference products, although this effectiveness varies depending on the tests. Several studies have demonstrated the strong antioxidant potential of *A. muricata*, suggesting that this plant could be a valuable source of antioxidants [34]. This activity is primarily linked to the presence of phenolic compounds [35, 36]. Flavonoids and phenolic acids exert their antioxidant action mainly by scavenging free radicals [37]. The mechanism involves the transfer of a hydrogen atom from the hydroxyl group of the polyphenol to the peroxyl radical, thus forming an HO bond with an electron [33]. However, the antioxidant effectiveness of phenolic compounds decreases in the presence of solvents capable of forming hydrogen bonds with these compounds [33].

Furthermore, the antioxidant activity of *A. muricata* has been confirmed by various in vitro tests, such as DPPH, ABTS^+^, ORAC, and FRAP [25, 33], highlighting the potential of this plant to protect against oxidative damage. This antioxidant property is essential, as it helps reduce inflammation and pain by limiting the formation of free radicals, which activate inflammatory pathways [37]. This powerful antioxidant effect is crucial for combating oxidative stress, which is responsible for various inflammatory and degenerative pathologies, as well as cellular aging.

Toxicity studies show that the *A. muricata* extract is well tolerated at high doses. Oral administration of the aqueous extract to mice revealed an LD_50_ greater than 5000 mg/kg, indicating low toxicity [37]. These findings are consistent with previous studies demonstrating the safety of graviola extract even at high doses [38, 39]. Additionally, intraperitoneal administration of the hydroalcoholic extract of the leaves in Swiss albino mice resulted in an LD_50_ of 1091.7 mg/kg [40].

However, doses higher than 5 g/kg may lead to adverse effects, particularly on the kidneys, whereas doses of 1 g/kg exhibit hypoglycemic and hypolipidemic effects [38]. Moreover, extracts enriched with acetogenins are more toxic than crude extracts [39]. The results obtained by Agu and Okolie [41] showed that extracts from the leaves and fruits of *A. muricata* had an LD_50_ of 1918.33 mg/kg, whereas both the stem bark and root bark had an LD_50_ greater than 5000 mg/kg. Quilez et al. [42] also conducted an acute toxicity study on the leaves of *A. muricata* in male Swiss albino mice aged 5 weeks. No signs of toxicity or mortality were observed during the 48 h following the administration of the extract at doses of 250, 500, and 1000 mg/kg, and no macroscopic lesions were detected. Furthermore, the extract showed no toxicity on murine peritoneal macrophages, as confirmed by a mitochondrial reduction MTT assay.

Although the extract is generally well tolerated, precautions are necessary due to the presence of acetogenins, which are potentially neurotoxic compounds in case of prolonged ingestion at high doses [10]. However, the review conducted by Chan et al. [19] suggests that *A. muricata* has a favorable safety and tolerability profile. Available studies indicate that the use of *A. muricata* extract at moderate doses is safe for treating various disorders, particularly those related to inflammation and oxidative stress. The management of pain is often linked to the control of inflammation, and *A. muricata* has shown notable antinociceptive and anti‐inflammatory effects, both centrally and peripherally, often surpassing those of reference substances. Tests using the hot plate, writhing, formalin, and carrageenan‐induced edema have revealed different aspects of these activities. The hot plate test highlighted a central antinociceptive activity [43], whereas the writhing test showed a more modest peripheral analgesic action [44]. The analgesic effect of *A. muricata* extract is likely mediated by several mechanisms, including the inhibition of the release of pro‐inflammatory mediators such as histamine, serotonin, and prostaglandins. The formalin test demonstrated a two‐phase nociceptive activity: an initial neurogenic phase mediated by substance P and bradykinin, followed by an inflammatory phase involving histamine, serotonin, prostaglandins, and bradykinin [45]. Medications acting on the central nervous system inhibit both phases, whereas those with peripheral action only inhibit the late phase. Inflammation induced by carrageenan demonstrated that the graviola extract significantly reduces edema compared to acetylsalicylic acid (ASA) [46].

Moreover, the involvement of opioid and α2‐adrenergic receptors in the modulation of pain by the extract has been demonstrated [47, 48], although no antagonism by haloperidol, a dopaminergic antagonist, was observed, suggesting that this effect does not involve this system. A role for potassium channels has also been proposed, as they are blocked by glibenclamide [47]. These mechanisms highlight the interest of *A. muricata* in the integrated treatment of pain and inflammation.

On the other hand, the extract of *A. muricata* exhibits both central and peripheral antinociceptive effects, as well as a potent anti‐inflammatory effect. Numerous studies report these properties for the aqueous and ethanolic extracts of *Annona muricata* [49, 50]. The extract demonstrated anti‐inflammatory activity comparable to that of indomethacin, a non‐steroidal anti‐inflammatory drug, with effectiveness observed during both the neurogenic and inflammatory phases [51]. This activity is likely due to the inhibition of cyclooxygenase (COX) and lipoxygenase (LOX) enzymes, as well as the presence of flavonoids and phenolic compounds [52]. Additionally, the inhibition of lipid peroxidation by the antioxidant compounds of *A. muricata* contributes to the reduction of inflammation, thereby enhancing its potential as an anti‐inflammatory therapeutic agent and protector against oxidative damage. In terms of antinociceptive activity, the present findings are consistent with those of Hamid et al. [53]. The latter researchers observed significant analgesic effects in models of abdominal writhing induced by acetic acid and of pain induced by formalin following administration of an extract of *A. muricata* leaf ethanolic solution.

With regard to the potential anti‐inflammatory effects of the extract, it was found to exhibit the capacity to inhibit the action of inflammatory mediators, which included TNF‐α, IL‐6, and nitric oxide, in a variety of in vitro models. For instance, Laksmitawati et al. documented a 46.8% inhibition of TNF‐α in LPS‐induced RAW264.7 cells following treatment with 50 µg/mL of *A. muricata* leaf extract [54]. Moreover, Quilez et al. demonstrated a dose‐dependent reduction in paw edema in mice induced with carrageenan, with a 52.7% decrease observed at a dose of 500 mg/kg of the extract [42]. Moghadamtousi et al. [55] also observed significant anti‐inflammatory activity in a study on wound healing, with an increase in HSP70 [56]. Additionally, Chan and Moore [57] reported that the extract had significant anti‐arthritic activity in rats with induced arthritis, reducing edema and suppressing levels of TNF‐α and IL‐1β, with effects greater than those of high‐dose indomethacin. Finally, a study conducted by Mutakin et al. [9] revealed that a high dose of 1 g/kg of *A. muricata* could reduce the number of dopaminergic neurons in rats, likely due to the presence of acetogenins derived from annonacin. In accordance with the findings of the aforementioned study, our own research findings are in alignment with those previously reported.

## 3 Conclusions

*A. muricata* stands out for its multiple therapeutic properties, particularly its antioxidant, antinociceptive, and anti‐inflammatory effects. Its rich chemical composition, notably in flavonoids and polyphenols, is responsible for these benefits, whereas toxicity studies confirm its safety at moderate doses. Thanks to these qualities, *A. muricata* shows promising potential for the treatment of inflammatory and painful diseases, while minimizing the side effects associated with the long‐term use of conventional anti‐inflammatory drugs. Its favorable safety profile makes it an excellent candidate for safe and effective therapeutic use, provided that recommended doses are adhered to in order to avoid any potential toxic effects.

## 4 Experimental Section

### 4.1 Plant Materials

Leaves of *A. muricata* L. were imported from Indonesia by the soursop company “Leaves.” Voucher samples representing AML 19‐04 were identified by Professor A. Ouhammou and were deposited in the Herbarium of the Faculty of Sciences Semlalia, Marrakesh, Morocco. The plant material was initially dried in the shade at room temperature (23°C), after which it was washed with water and reduced to a very fine powder. The powder was extracted with distilled water (1 g/10 mL) under stirring for 12 h by cold maceration. The aqueous extract was then centrifuged (1200 rpm), filtered, and lyophilized in a Christ apparatus (Sigma Aldrich). The extract was stored in a freezer at a temperature of −20°C until required for use.

### 4.2 Phytochemical Study

#### 4.2.1 Total Phenolic Content

The TPC of the extract was determined using a modified Folin–Ciocalteu method [58]. In this procedure, 0.4 mL of the diluted extract was mixed with 1.5 mL of the Folin–Ciocalteu reagent. Then, 1.6 mL of a 7.5% sodium carbonate solution was added. The mixture was kept in the dark at room temperature for 2 h, after which the absorbance was measured at 765 nm. Gallic acid was used as the standard reference, and the results were expressed as milligrams of gallic acid equivalent per gram of dry weight (mg GAE/g DW).

#### 4.2.2 Total Flavonoid Content

The quantification of TFC in the extract was conducted using a method outlined by Zhishen et al. [59]. In this procedure, 200 µL of the extract was first diluted with 1 mL of distilled water. Then, 60 µL of 5% NaNO_2_ and 60 µL of 10% AlCl_3_ were added to the solution. After 5 min of incubation, 400 µL of 1 M NaOH was introduced into the mixture. The absorbance was then measured at 510 nm. The TFC was expressed as milligrams of catechin equivalents per gram of dry matter (mg CE/g DM).

#### 4.2.3 Total Condensed Tannins

The content of condensed tannins was determined using a method previously described by Aitbaba et al. [60]. Briefly, 400 µL of diluted samples were mixed with 3 mL of a 4% methanol vanillin solution and 1.5 mL of concentrated HCl. After 15 min of incubation, the absorbance was measured at 500 nm. The total condensed tannin content was quantified and expressed as milligrams of catechin equivalent per gram of dry matter (mg CE/g DM).

#### 4.2.4 HPLC Analysis

For the identification of phenolic and flavonoid compounds in AE, the samples were first filtered through Whatman filter paper no. 42 and then through 0.22 mm membrane filters (Millipore). The separation of these compounds was carried out using HPLC. A reversed‐phase RP‐18 column (250 mm × 4.6 mm, 5.0 µm) from Agilent Technologies, protected by an Agilent Technologies RP‐18 precolumn (10 mm × 4.6 mm), was used for chromatographic separation. Both columns were maintained at 25°C in the oven. The HPLC system comprised a Shimadzu (Japan) SCL‐10A series pumping system, SIL‐10AD automatic injector, and an SPD 10A UV–visible detector set with a detection range from 200 to 700 nm. Data collection and analysis were performed using the Shimadzu LC Solution chromatography data station software [61, 62].

The separation utilized two solvents with a constant flow rate of 0.1 mL/min and an injection volume of 10 µL. Solvent A consisted of an isocratic mixture of acetonitrile (5%) and water (95%), whereas solvent B was a phosphate buffer solution at pH 2.6. All solvents were of HPLC grade. Phenolic compounds were identified by comparing their retention times with those of standards, which included gallic acid, syringic acid, tyrosol, rutin, catechin, and quercetin.

### 4.3 Antioxidant Activity

#### 4.3.1 DPPH Free Radical Scavenging Activity

The antioxidant activity of the extract was evaluated using the DPPH assay, following the method outlined by Mansouri et al. [63] [60], with modifications by Baslam et al. [64]. In brief, 1.5 mL of a methanolic DPPH solution (6 × 10^−5^ M) was mixed with 60 µL of the extract at various concentrations (1, 2, 4, 6, and 8 mg/mL) of AEMV. The mixture was then kept in the dark at room temperature for 30 min. After incubation, absorbance was measured at 515 nm. A negative control, consisting of 1.5 mL of the DPPH solution and 60 µL of methanol, was used. Positive controls included BHT and quercetin. The percentage of inhibition was calculated using the following formula:

$$\mathrm{Inhibition}\;\left(\%\right)=\left[\left(A_{\mathrm{control}}-A_{\mathrm{sample}}\right)\;/\;A_{\mathrm{control}}\right]\times 100$$

where *A*_control_ represents the absorbance of the control, and *A*_sample_ denotes the absorbance of the test compound.

The concentration of the sample that causes 50% inhibition (IC_50_) was ascertained from a graph illustrating the percentages of inhibition plotted against the sample concentrations.

#### 4.3.2 Ferric‐Reducing Ability Power (FRAP)

The FRAP test, on the basis of the Oyaizu method [65], involves preventing the formation of Fe(II)–ferrozine complexes during the incubation of the sample with ferrous iron. In practice, a mixture consisting of 1 mL of distilled water, 2.5 mL of phosphate buffer (0.2 M, pH 6.6), and 2.5 mL of potassium ferricyanide (K_3_[Fe(CN)_6_]) (1%) was combined with 0.5 mL of various sample solutions. After a 30‐min incubation period, 2.5 mL of distilled water, 2.5 mL of 10% trichloroacetic acid, and 0.5 mL of FeCl_3_ were added to the mixture. The absorbance was then measured at 700 nm. Quercetin and BHT were used as positive controls.

### 4.4 β‐Carotene Bleaching Test

In this test, antioxidant capacity is determined by measuring the inhibition of oxidative degradation of β‐carotene (discoloration) by linoleic acid oxidation products. The standard antioxidant is BHT.

β‐Carotene of 0.5 mg is dissolved in 5 mL of chloroform. This solution of 1 mL is then added to a beaker previously containing 200 mg of Tween 40 and 20 mg of linoleic acid. After evaporating the chloroform, 50 mL of distilled water is added, and the resulting emulsion is shaken vigorously. The β‐carotene/linoleic acid emulsion of 2.5 mL is added to 100 µL of the extract solution or the synthetic antioxidant BHT at various concentrations in tubes. Absorbance was measured immediately at 470 nm, corresponding to *t* = 0 min, against the blank containing the emulsion without β‐carotene only for the negative control.

The antioxidant activity (%) of the extract was evaluated in terms of β‐carotene bleaching using the following formula:

$$I\;\%\;=[\left(AA\left(120\right)\;-\;AB\left(120\right)\right)/\left(AB\left(0\right)\;-\;AB\left(120\right)\right]\;\times \;100$$

where *I*% is percentage inhibition, and *AA*(120) and *AB*(120) represent the absorbance in the presence of the extract (antioxidants) and the control, respectively, at 120 min. *AB*(0) represents the absorbance of the control at 0 min.

### 4.5 Animal Study

#### 4.5.1 Animals

Male Swiss mice (25–35 g) and Wistar albino rats (150–200 g) were sourced from the animal care unit of the Faculty of Science Semlalia, Cadi Ayyad University, Marrakech, Morocco. These animals were kept at a stable ambient temperature (22°C ± 2°C) with a 12‐h light/dark cycle and had unrestricted access to food and water. Every effort was made to minimize potential animal suffering.

#### 4.5.2 Acute Toxicity

The toxicity examination was conducted using Swiss albino mice. The animals were randomly assigned to one control lot and four treated lots. The treated groups received the plant extract orally at different doses. The doses administered were 1000, 2000, and 5000 mg/kg. Following the administration of the concentrate, the animals were observed for any changes in behavior or clinical signs, as well as for mortality. The observation period commenced immediately following the administration of the concentrate and continued for a total of 4 h. Thereafter, the animals were observed every hour for the subsequent 24 h. Furthermore, for the subsequent 48 h, observation was conducted at 6‐h intervals. The treated groups were observed for 1 week to determine whether any further changes occurred.

#### 4.5.3 Hotplate Test

This test consists of studying the animal's reaction following short‐term thermal nociceptive stimulation. The animal is placed on a plate heated to 55°C ± 1°C. The latency time it takes to lick one of its paws or to jump was recorded and considered as the reaction time. The duration of this test must not exceed 20 s in order to avoid damaging the skin tissue of the paws [66].

#### 4.5.4 Writhing Test

The antinociceptive activity was investigated via the writhing test engendered, in mice, by acetic acid at a concentration of 0.6% (0.1 mL/10 mg, [intraperitoneal]). The various doses were given orally 45 min earlier than the nociceptive agent. Five minutes after the acid injection, the number of writhes and stretching movements (contraction of the abdominal musculature and extension of hind limbs) was counted for a period of 30 min. ASA (200 mg/kg body weight) was used as pharmacological reference [67]. Antinociceptive activity was expressed as the reduction in the number of abdominal constrictions between the control animals and the mice pre‐treated with compounds.

#### 4.5.5 Formalin Test

Formalin is an inflammatory molecule producing tissue damage. It causes a biphasic response of licking the injured paw following the injection and stimulation of the nociceptors. A late and prolonged nociceptive phase following the development of inflammation. The test described by Shannon and Lutz [68] consists of injecting a diluted formalin solution (20 µL, 2% per mouse) into the sole of the mice's hind leg. Afterward, the animals are placed in a transparent Plexiglas enclosure and the licking time of the injured paw is measured for 30 min. The nociceptive response is represented by the accumulation of licking time in the early phase (5–10 min) and in the late phase (15–30 min).

#### 4.5.6 Elucidation of the Mechanisms Underlying Antinociceptive Activity Using Antagonists

To elucidate the mechanisms of the antinociceptive activity of *A. muricata*, we used inhibitors of different pathways: naloxone, atropine, glibenclamide, haloperidol, and yohimbine. The doses of antagonists and other drugs were selected on the basis of data from the literature [69].

Naloxone was injected at a dose of 2 mg/kg (i.p.) 15 min before the administration of the test substance (aqueous extract of Graviola) at the highest dose, 500 mg/kg. Forty‐five minutes later, the formalin test and the hot plate test were initiated. The reference drugs, atropine (5 mg/kg, i.p.), glibenclamide (10 mg/kg, i.p.), haloperidol (20 mg/kg, i.p.), and yohimbine (1 mg/kg, i.p.), were injected 15 min before the oral administration of the test substances at the highest dose. Sixty minutes later for glibenclamide and 45 min for haloperidol and yohimbine, the writhing test was initiated as previously described.

### 4.6 Anti‐Inflammatory Activity Test

#### 4.6.1 Xylene‐Induced Ear Edema

The test aims to determine the supposed anti‐inflammatory effect of certain chemical compounds following the induction of edema on the internal and external surface of the ear [70]. A total of 30 mice used were divided into five groups of 6 mice each. Group I was treated with physiological serum (0.9% NaCl) as a control, and Groups II–IV were treated with AEAM extract at 250, 500, and 1000 mg/kg, respectively. The treatment was carried out 30 min before the test. Group V was treated with indomethacin (10 mg/kg) as a positive control. Fifteen minutes after xylene application, the animals were sacrificed by cervical dislocation in accordance with ethical standards. The right and left ears were isolated, and 5 mm sections were taken and weighed. Ear edema was assessed by calculating the weight difference between the right and left ear sections from the same animal. Ear sections were fixed in 10% formalin and cut into 4 µm thick sections, which were stained with hematoxylin and eosin for analysis.

The percentage of edema inhibition was calculated according to the following formula: inhibition (%) = difference in ear weight (control)/difference in ear weight (treated)/difference in ear weight (control) × 100%.

#### 4.6.2 Carrageenan‐Induced Rat‐Paw Edema

The evaluation of anti‐inflammatory activity was conducted by measuring carrageenan‐induced paw edema in rats, following the method described in a previous study [71]. Wistar rats were divided into five groups: control, AEAM at doses of 250, 500, and 1000 mg/kg, and indomethacin at 10 mg/kg. The extract and indomethacin were administered 45 min before the injection of 0.1 mL of carrageenan (1% in physiological saline) into the subplantar region of the paw. Subsequently, the paw volume was measured using a digital caliper before carrageenan injection at 1–4 h post‐injection. The obtained results were compared with those of rats receiving indomethacin orally (10 mg/kg).

The inhibition (%) of edema was determined using the following formula:

$$\mathrm{Inhibition}\;\left(\%\right)=\left[\left(V_{\mathrm{control}}-V_{\mathrm{sample}}\right)/V_{\mathrm{control}}\right]\times 100$$

where *V*_sample_ represents the volume (in mL) of the paw of the mouse receiving the different treatments at the corresponding time point, and *V*_control_ represents the volume of the paw of the control group mouse at the same time point.

### 4.7 Statistical Analysis

The results were expressed as mean ± standard errors of the mean (SEM). The comparison between the various groups was carried out with one‐way analysis (one‐way analysis of variance [ANOVA]), and the repeated measures ANOVA model was monitored by Tukey's post hoc test. A value of *p* < 0.05 was assumed to be statistically significant.

## Author Contributions

**Sara Oufquir**: conceptualization, writing – original draft, methodology. **Fatimazahra Agouram**: methodology. **Hamid Kabdy**: conceptualization, writing – original draft. **Mehdi Ait Laaradia**: writing – original draft, methodology. **Khadija Oubella**: writing – original draft, methodology. **Baslam Abdelmoniim**: writing – original draft. **Stefania Garzoli**: writing – review and editing, supervision. **Abderrahman Chait**: validation, writing – original draft, supervision. All authors have participated sufficiently in the work and agreed to be accountable for all aspects of the work.

## Ethics Statement

All animal procedures complied with the European Council Directive 2010/63/EU on the protection of animals used for scientific purposes. Ethical approval for the experiments was granted by the Institutional Ethics Committee of the Faculty of Sciences at Cadi Ayyad University in Marrakech, Morocco, in accordance with the guidelines of the Committee for the Regulation and Oversight of Animal Experimentation and Ethics. The study was assigned protocol code BAM1212/04/23, with approval obtained in April 2023.

## Conflicts of Interest

The authors declare no conflicts of interest.

## Data Availability

The data that support the findings of this study are available from the corresponding author upon reasonable request.
